# Enhancing Anterior Esthetic Zone Implant Placement Through Bone Manipulation Techniques: A Case Series

**DOI:** 10.7759/cureus.65559

**Published:** 2024-07-28

**Authors:** Indu Raj, Harinee A, Anjana s Raj, Arvind K Uikey, Femitha Syed

**Affiliations:** 1 Prosthodontics, Government Dental College, Kottayam, Kottayam, IND; 2 Prosthodontics, Chettinad Dental College and Research Institute, Chennai, IND; 3 Prosthodontics, Thalassery Co-operative Hospital, Calicut, IND; 4 Prosthodontics, Shahid Bhagat Singh District Hospital Balaghat, Balaghat, IND

**Keywords:** socket shield, osseodensification, expansion, buccolingual width, implants

## Abstract

Replacement of missing teeth using dental implants has become the most frequently performed procedure. Following tooth loss, buccolingual bone width decreases significantly compromising the successful placement of dental implants. Various treatment modalities have been advocated in scenarios with insufficient buccolingual bone width like narrow diameter implant placement, horizontal guided bone regeneration, ridge splitting technique, and osseodensification. Maxillary anterior tooth loss is of prime esthetic concern, which needs immediate attention. Guided bone regeneration is the gold standard for patients presenting for rehabilitation in the anterior maxilla with inadequate buccolingual bone width. However, bone grafting techniques require longer treatment time; hence, various other techniques like lateral bone expansion, osseodensification, or socket shield technique are sought. This case series presents successful rehabilitation of the maxillary anterior esthetic zone with dental implants using various bone manipulation techniques, including lateral bone condensation, socket shield technique, and osseodensification.

## Introduction

In recent years, the replacement of missing teeth using dental implants has become a predictable and successful treatment modality. Predictable replacement of teeth using dental implants depends on successful osseointegration of the implant to alveolar bone for which a sufficient volume of healthy bone must be available. Apart from the quality and vertical height of bone, buccolingual bone width is a prime factor to be considered for a predictable outcome. According to Buser et al. [1], at least 1 mm of bone volume should be present on both the buccal and lingual aspects of the implant to achieve a long-term favorable prognosis.

Maxillary anterior teeth are considered to be one of the most important esthetic facial features by most of the patients irrespective of low or high smile line. Trauma or periodontal or endodontic disease resulting in tooth loss is often accompanied by severe tissue deficiencies in the anterior maxilla compromising facial esthetics in some patients [2]. Following tooth loss, the bucco-lingual alveolar ridge dimension decreases significantly, which can result in a loss of as much as 50% of the original bone width [3]. To place an implant of 3.5-4 mm diameter implant, there is a minimal requirement of 6-7 mm of bone width as stated in the literature. Frequently, cases of implant placement involve insufficient buccolingual width of the edentulous ridge. The methods used to resolve this issue are narrow implant placement, horizontal veneer block bone graft, horizontal guided bone regeneration (GBR), ridge splitting technique, and osseodensification.

Lateral bone expansion, first introduced by Hilt Tatum in 1970, prescribes the usage of special burs and has been a frequently followed technique, especially in the maxillary anterior region where the labial bone is usually inadequate. This technique involves the usage of “screw-type” burs and thread formers, which mimic the shape of an implant, in increasing diameters for lateral bone expansion and condensation, aiding in the placement of an endosseous dental implant of suitable diameter without damaging the available labial bone. These burs are used to widen the preparation site and not the depth [4]. These techniques are used as an alternative to block grafting to increase ridge width, allowing immediate placement of implants in narrow ridges.

The socket shield (SS) technique, a novel treatment modality that was ideated by Hurzeler in 2007, can be used in aesthetically challenging cases to preserve the post-extraction hard and soft tissues. The principle involves the preparation of the root of the tooth in such a manner that a fragment of the root is left in the socket to preserve the tissues in its physiological relation [5].

Osseodensification brought into the forum by Koutouzis et al. involves the usage of specially designed burs. When these burs are used in counterclockwise rotation with external irrigation (densifying mode), the dense compact bone tissue is condensed along the osteotomy walls. The swaying motion generates a rate-dependent stress, which in turn produces a rate-dependent strain and lets the irrigant flow to gently pressurize the bone walls. This combination facilitates increased bone plasticity and bone expansion. Thus, after implant placement, it is observed that there is an increase in immediate to early bone-to-implant contact, which occurs as a result of spring back effect and rebound of bone on the implant. Thus, implants placed after osseodensification have better primary stability [6].

Bone grafting and GBR is the most ideal procedure to increase the buccolingual bone width, and to some extent, the vertical height of the bone can also be increased. Autogenous bone grafting is the golden standard by which all techniques of osseous reconstruction must be evaluated. Extensive research has been focused on the development of suitable bone substitutes based on minerals, which are found in bone. Hydroxylapatite (HA) is one such major bone mineral. GBR, which was introduced for the treatment of vertical bone defects, has also been tried for minor augmentation procedures before dental implant placement. Membranes are frequently placed beneath bone grafts to achieve successful bone gain for implant placement [7].

A case series is presented here, with a case for each of the above-mentioned three techniques, which were all carried out in the anterior esthetic zone with a follow-up period ranging from six months to one year.

## Case presentation

Case 1: Bone expansion screw technique

**Figure 1:**
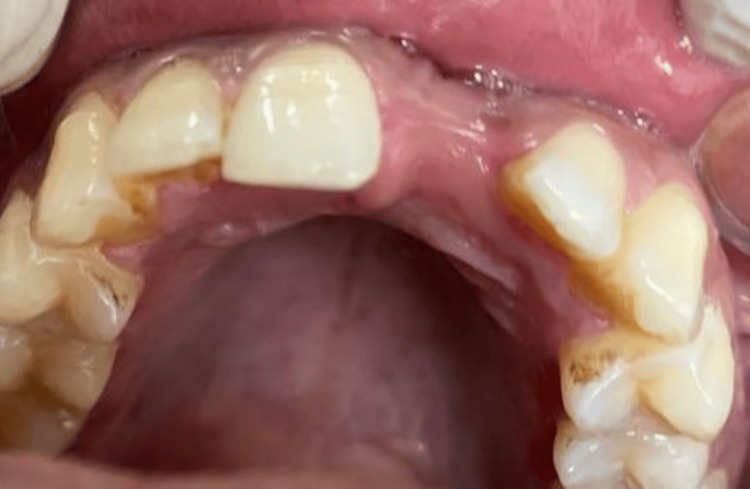
Case 1: Preoperative intra-oral image depicting narrow alveolar bone with buccal concavity and adequate soft tissue thickness in relation to missing 21

**Figure 2:**
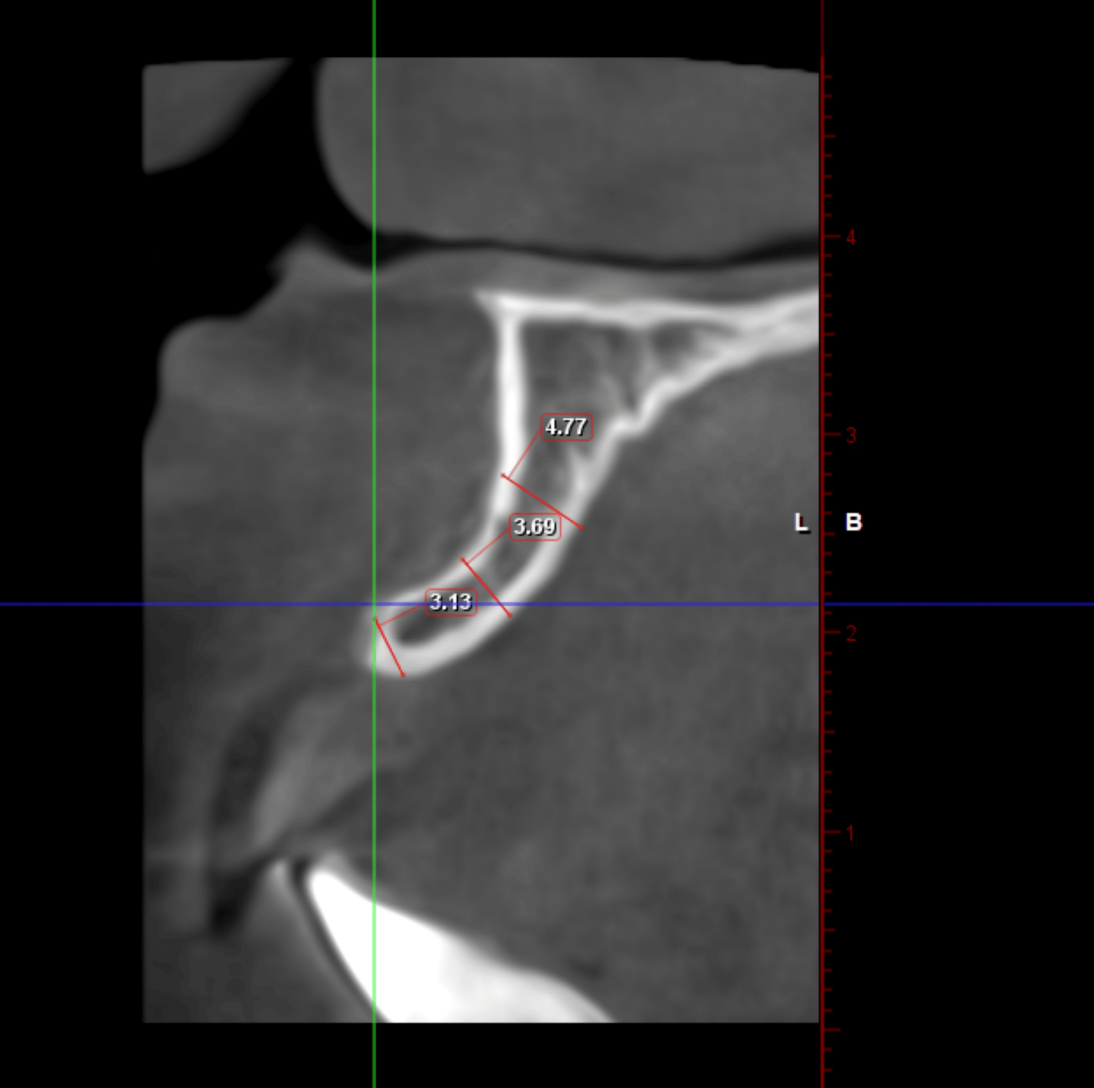
Case 1: Coronal section of CBCT image showing 3.13 mm of buccolingual bone at the crest, 3.69 mm bone at the middle third, and 4.77 mm bone apically denoting inadequate buccolingual bone for implant placement using conventional osteotomy CBCT: Cone beam computed tomography.

A 22-year-old female patient reported to the Department of Prosthodontics with the chief complaint of a missing upper front tooth (Figure 1). The treatment plan included prosthetic rehabilitation of 21 with an implant-supported crown. On examination of the cone beam computed tomography (CBCT) images of the patient, it was evident that buccolingual bone width was inadequate for implant placement (crest: 3.13 mm, middle: 3.69 mm, and apical: 4.77 mm) as shown in Figure 2.

**Figure 3:**
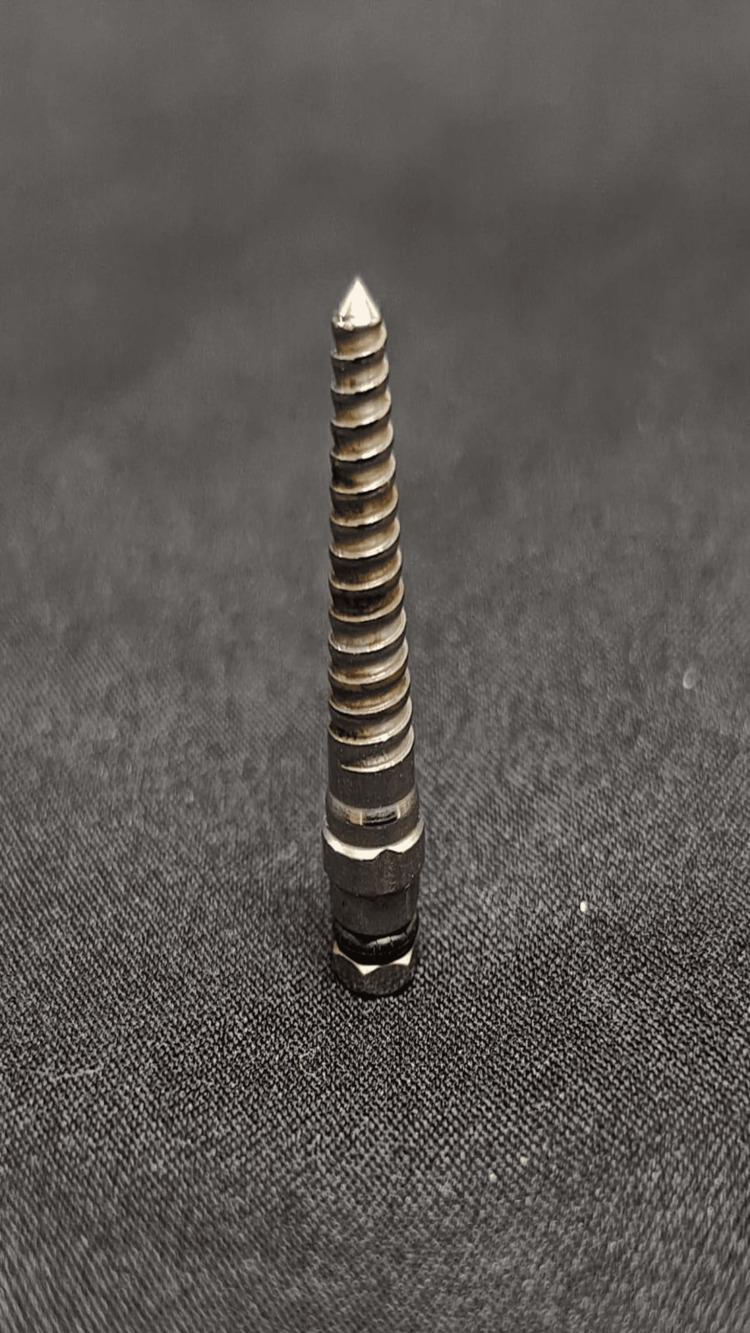
Case 1: Bone expansion screw/thread former used for lateral bone expansion in narrow ridges or ridges with buccal concavity, which is used sequentially with increasing diameters from 1.6 to 2.8 mm and 1.9 to 3.4 mm

**Figure 4:**
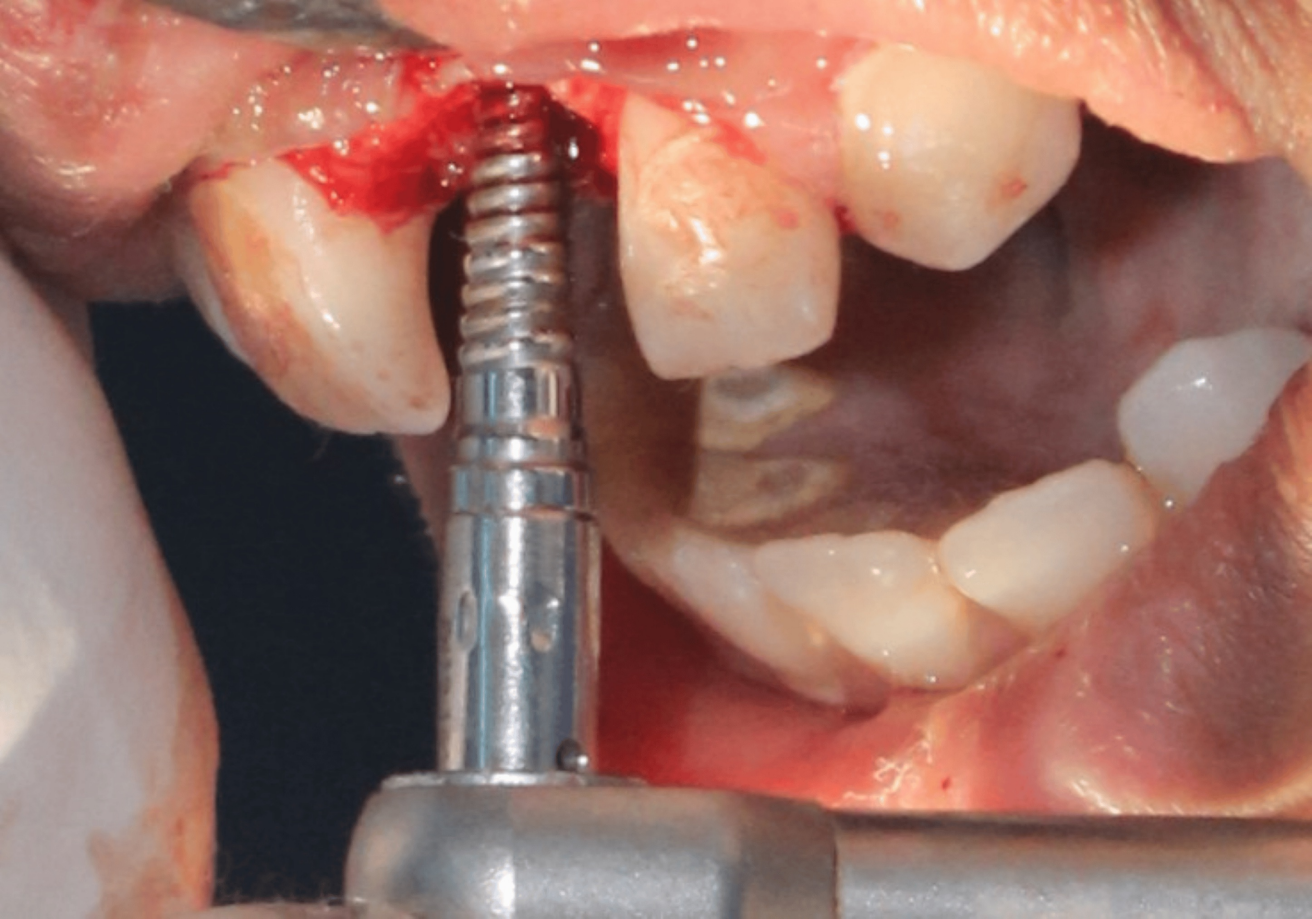
Case 1: Lateral bone expansion done using bone expansion screws of increasing diameters with the help of a ratchet that allows half of a turn at a time

To preserve the existing bone and prevent perforation or fracture of the labial bone, the implant procedure was performed using bone expansion screws (Figure 3), which took the shape of the implant to be placed. Bone expansion using screws was done sequentially with increasing diameters from 1.6 to 2.8 mm and 1.9 to 3.4 mm as depicted in Figure 4.

**Figure 5:**
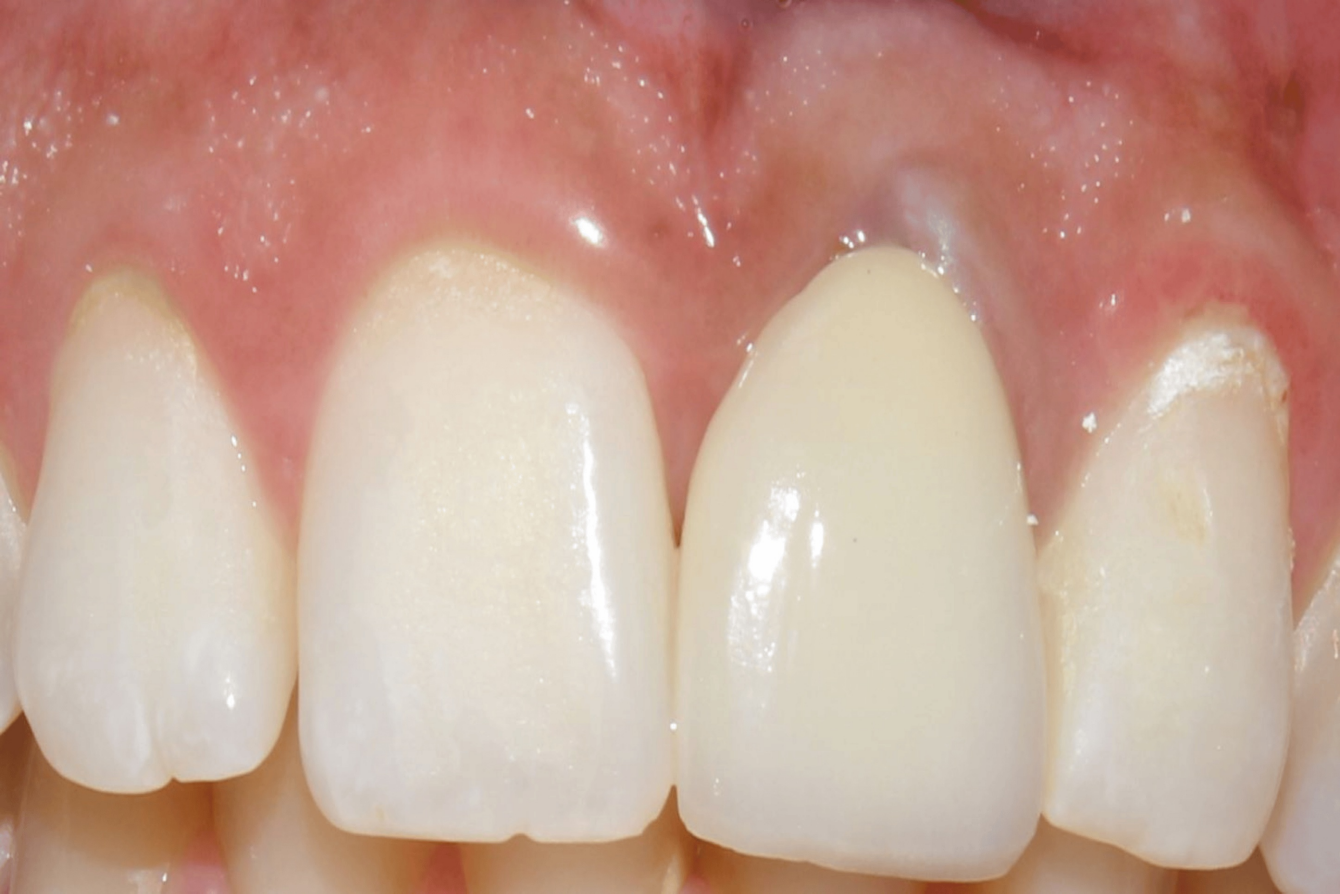
Case 1: After assessment of successful osseointegration, the prosthetic phase was done, and the implant with respect to 21 was loaded using a cement-retained crown as the screw access hole was labial

**Figure 6:**
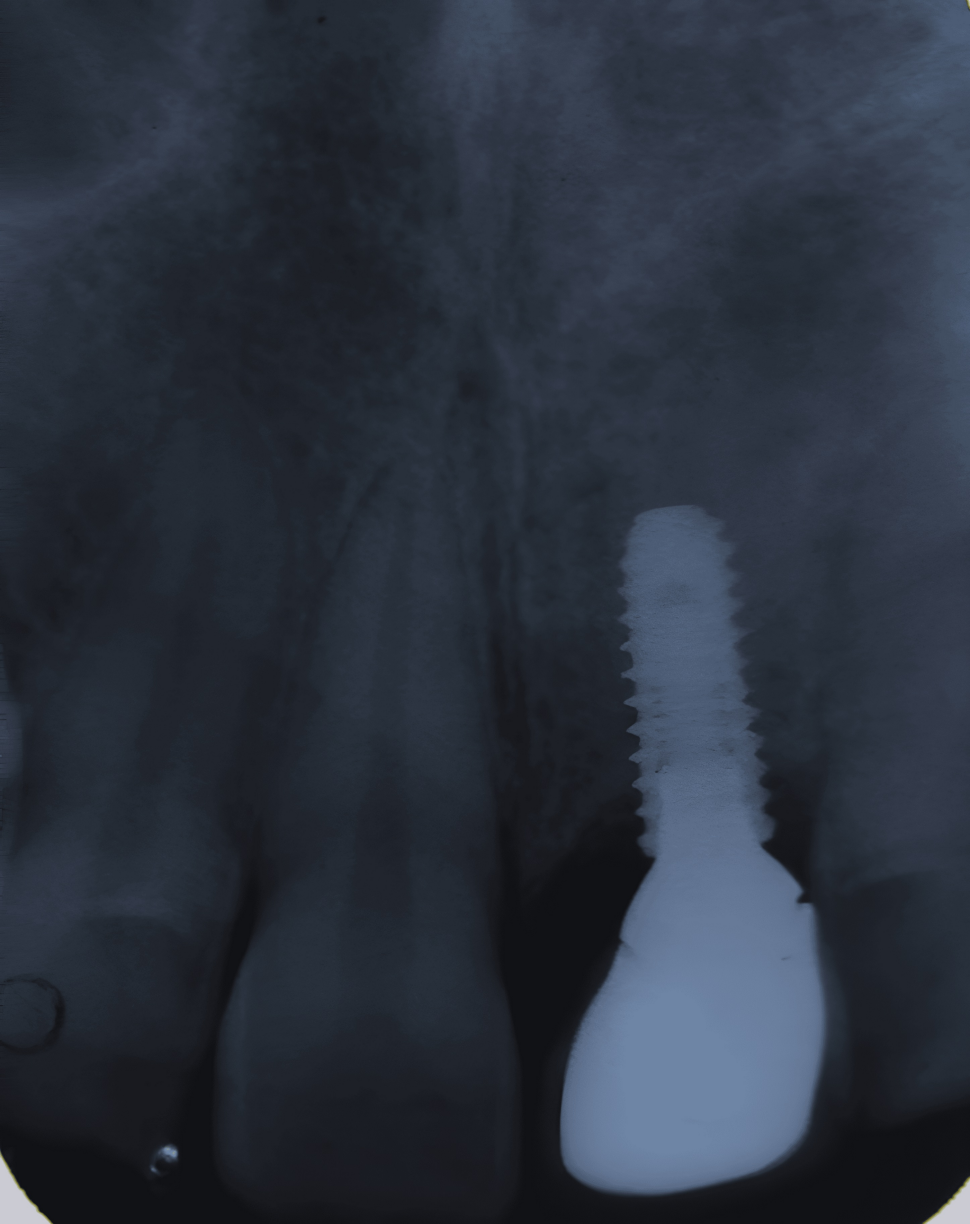
Case 1: Intra-oral periapical radiograph was taken for the patient at the follow-up visit after one year, showing mild bone loss of 0.2 mm, which is the expected amount of bone loss according to Albrektsson's criteria

Initially, thread formers are tightened with finger pressure. Then a ratchet that allows half of a turn at a time is used, which allows gradual horizontal expansion. Following expansion, the implant of dimension 3.5 x 10 mm was placed at the prepared site and sutured. A cement-retained crown was inserted after the assessment of successful osseointegration (Figure 5). Review appointments were scheduled at three months, six months, and one year. Intraoral periapical radiograph (IOPA) taken at the time of the one-year follow-up is depicted in Figure 6.

Case 2: Socket shield technique

**Figure 7:**
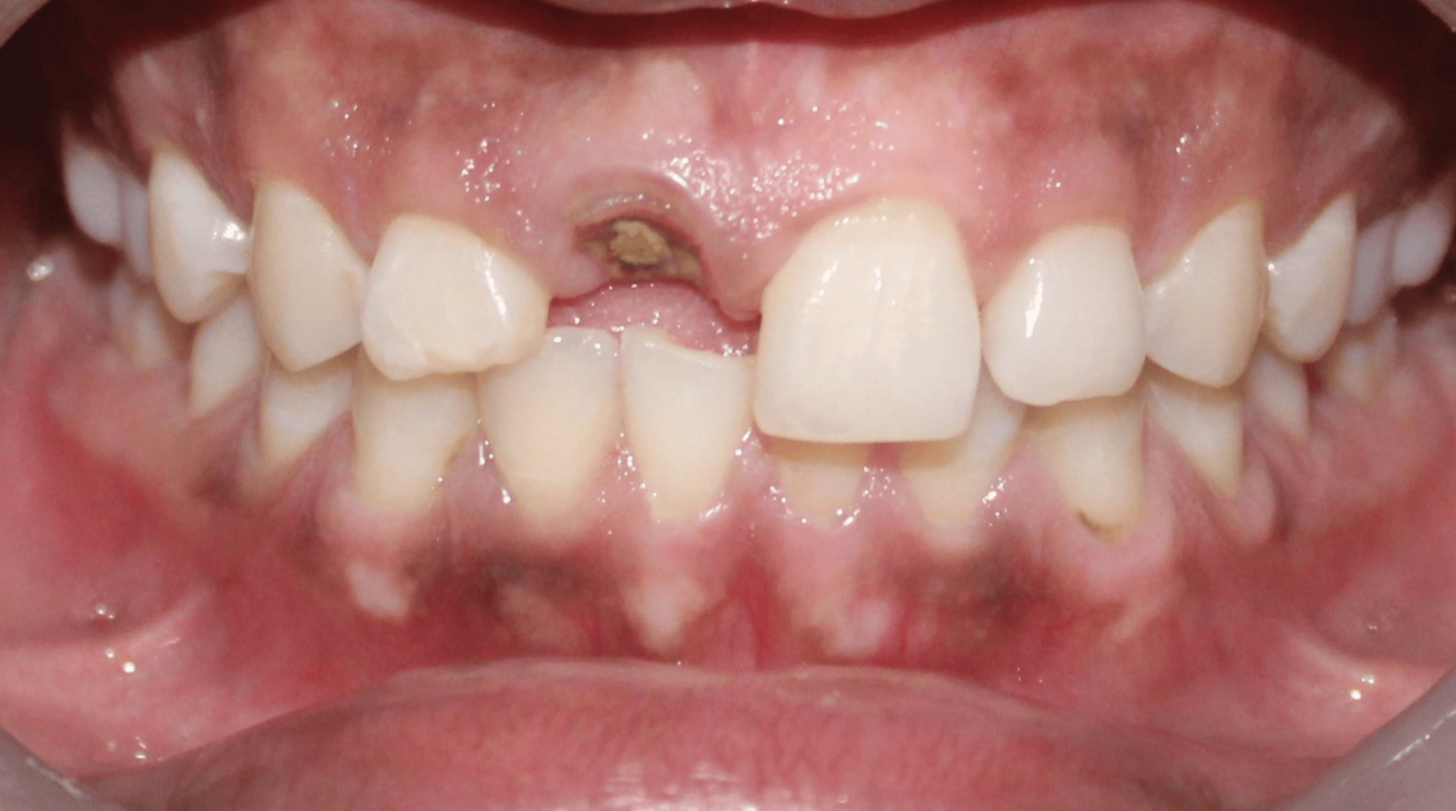
Case 2: Preoperative intra-oral image showing fractured 11 at the level of cementoenamel junction, with adequate soft tissue thickness but lacking the necessary ferrule for salvaging the tooth using post and core

**Figure 8:**
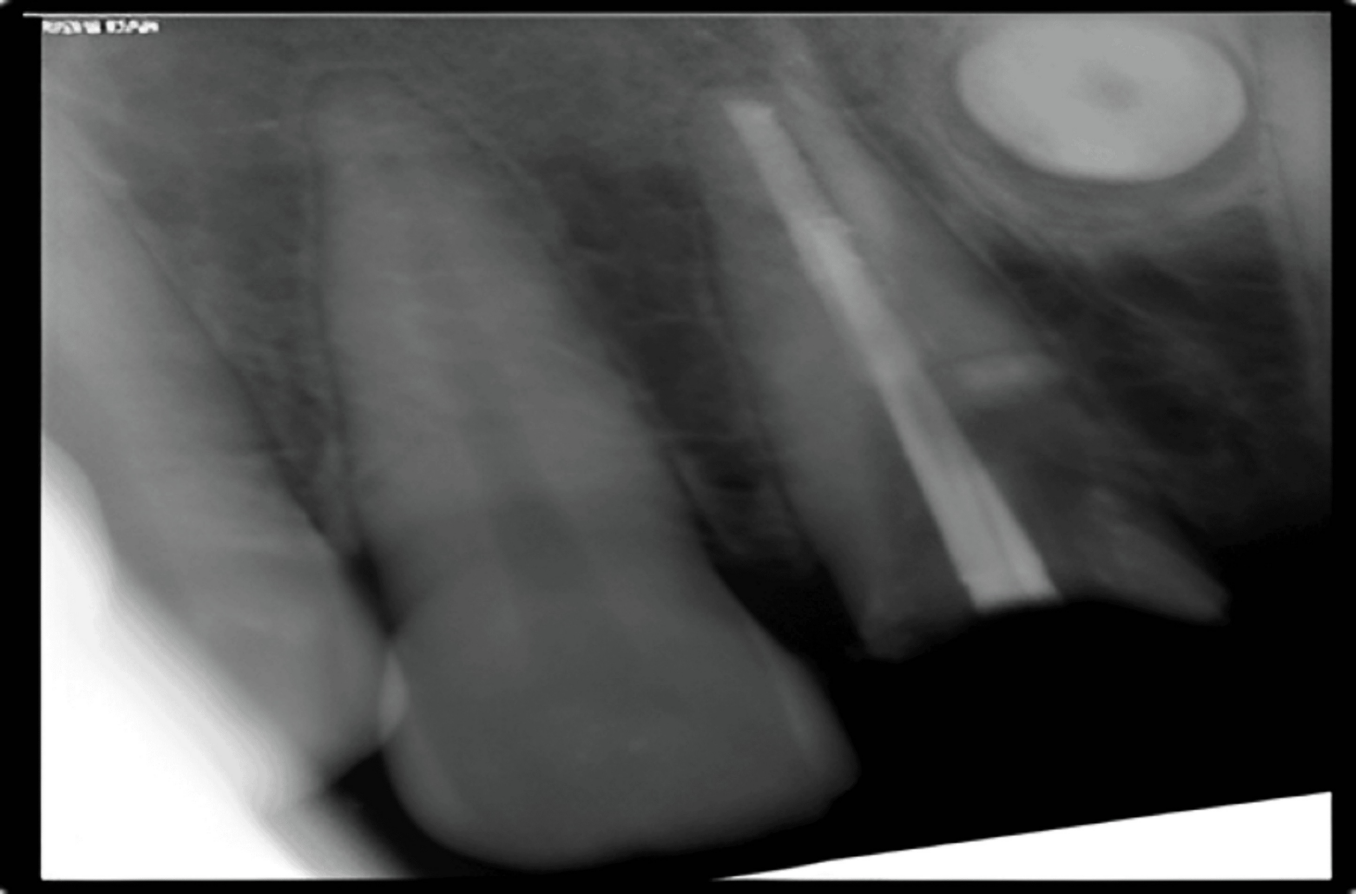
Case 2: Preoperative RVG image showing root canal treated 21 fractured at CEJ RVG: Radiovisiography; CEJ: Cementoenamel junction.

A 23-year-old patient reported for definitive treatment of a fractured upper front tooth. Although the tooth was root treated, after several years, it was fractured at the gingival level as depicted in Figures 7, 8.

**Figure 9:**
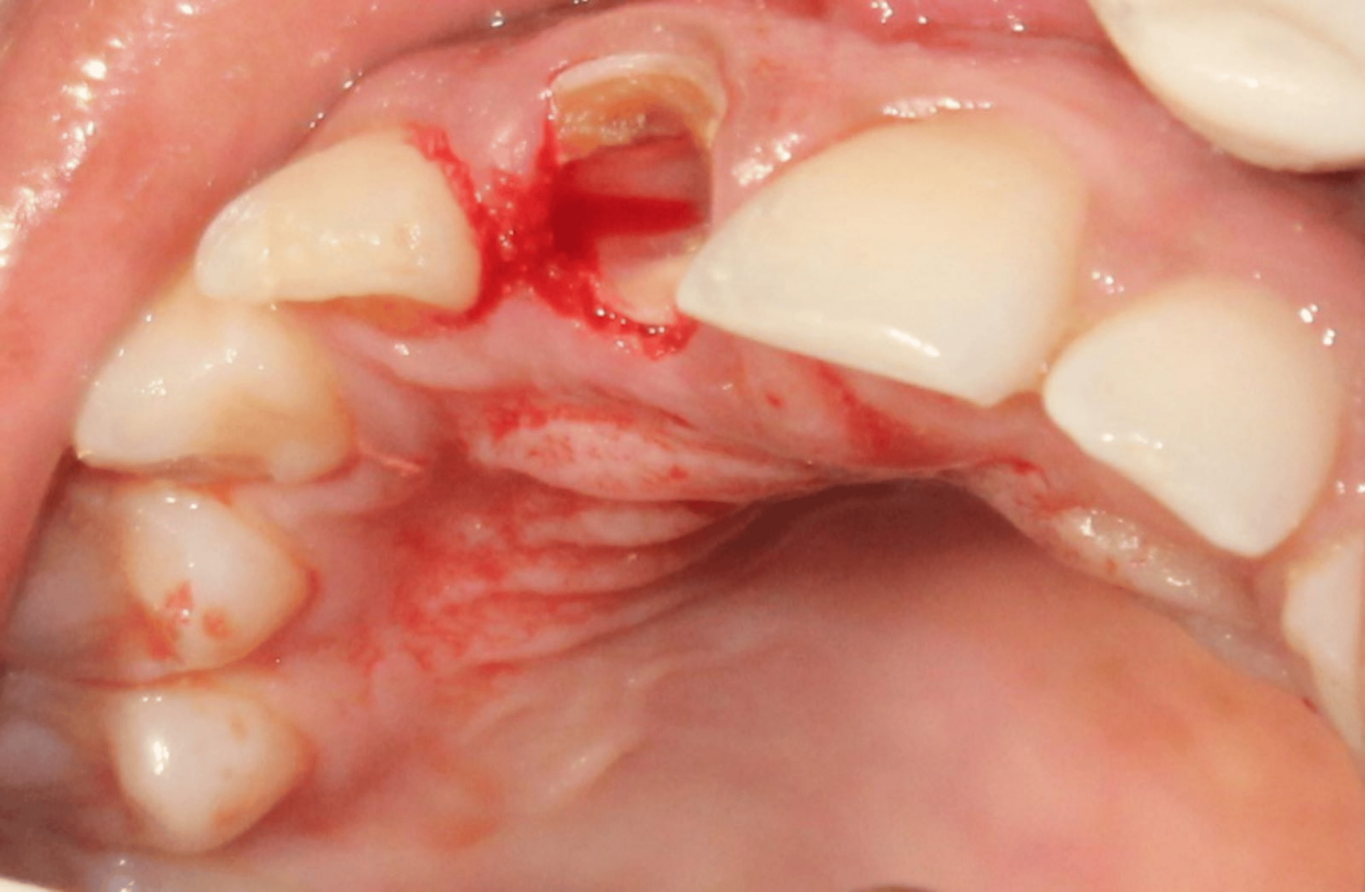
Case 2: Intra-oral image showing vertical sectioning of the root into buccal and palatal fragments using a long shank bur (Jull Dent-094A) of length 28 mm in a high-speed handpiece

**Figure 10:**
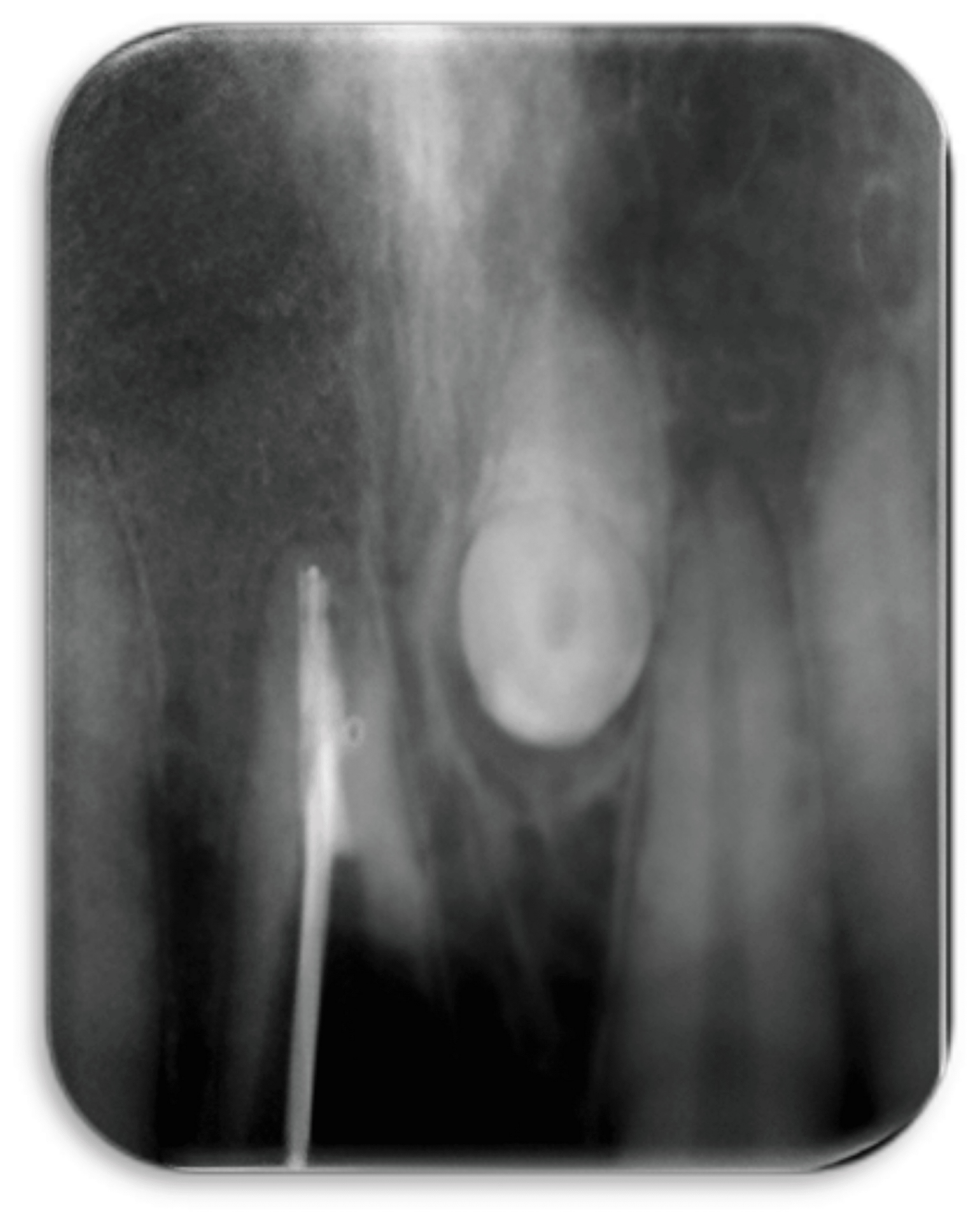
Case 2: RVG taken intra-operatively to assess the level of root resection RVG: Radiovisiography, digital x-ray.

In consultation with the patient considering costs, duration of treatment, and prognosis, implant therapy was opted for. The SS technique was planned for this patient to negate the effect of post-extraction ridge resorption. Preoperative CBCT indicated sufficient width palatal to the planned facial root section to accommodate a 3.5 x 13 mm implant. Following local anesthesia at the treatment site, the residual crown was reduced to a gingival level. A long shank bur (Jull Dent-094A) of 28 mm length was used in a high-speed handpiece to resect the root into facial and palatal halves (Figures 9, 10).

**Figure 11:**
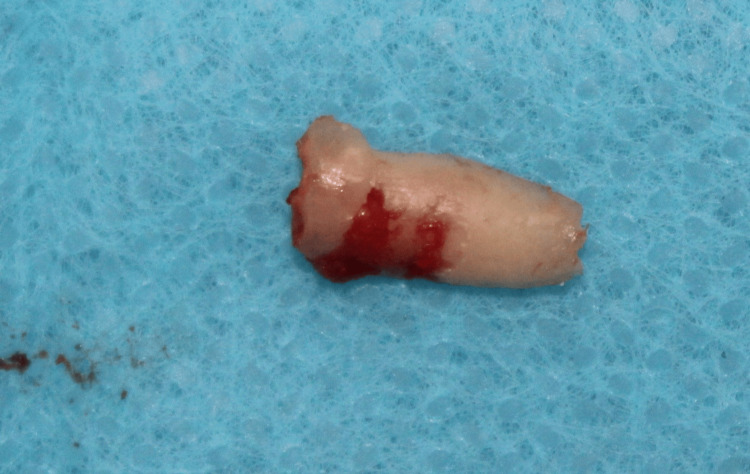
Case 2: After vertical sectioning, the palatal fragment along with the apex of tooth 11 was extracted with the help of periotome to preserve the buccal root and buccal bone Tooth number 11 = maxillary right central incisor.

**Figure 12:**
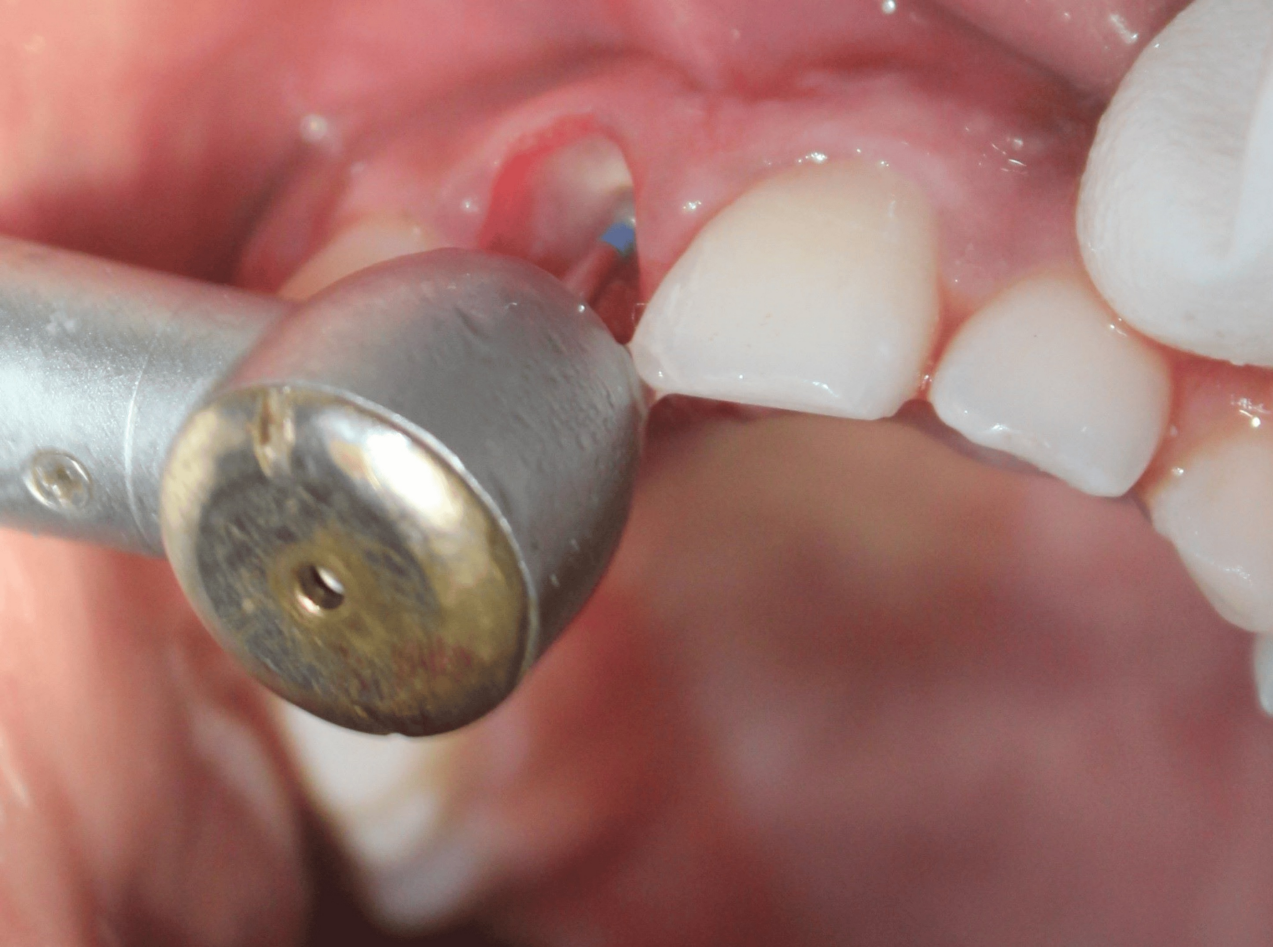
Case 2: The remaining labial shield was reduced coronally to 1 mm above the alveolar crest using a round bur in a high-speed handpiece, and bevel preparation was done

Following the sectioning of the root, the facial fragment was preserved to act as a shield, and the palatal fragment along with the apex was extracted (Figure 11). The remaining facial root section was then reduced coronally to 1 mm above the alveolar crest and thinned slightly to a concave contour as depicted in Figure 12.

**Figure 13:**
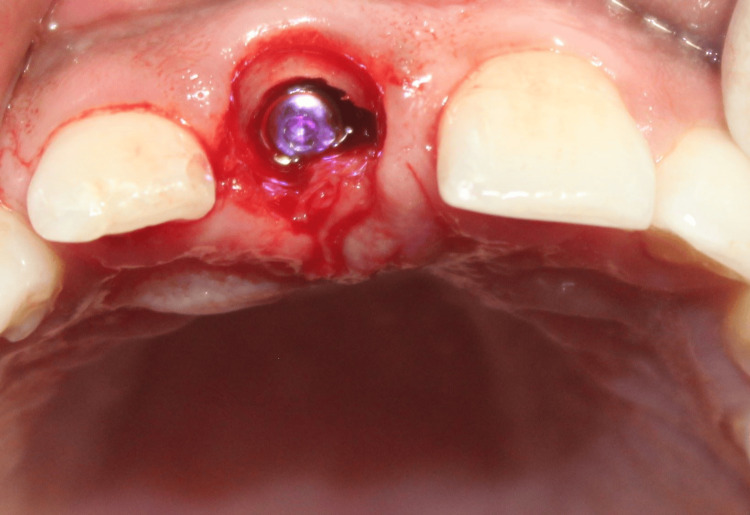
Case 2: Osteotomy was then sequentially prepared, and a 3.5 x 13 mm implant was inserted palatal to the socket shield

**Figure 14:**
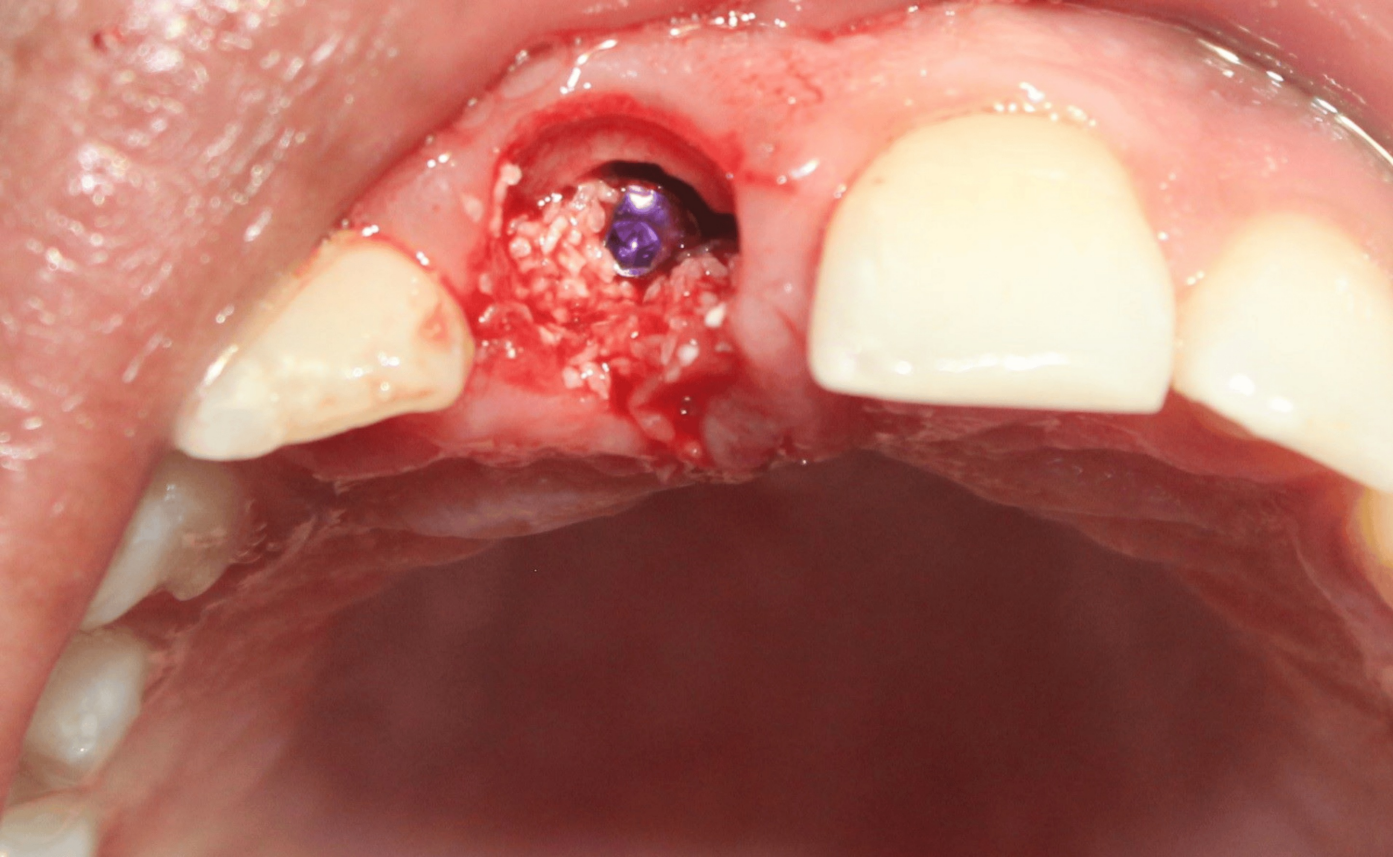
Case 2: The jump gap between the implant and the shield was filled with 100 mg of osseograft (xenogenic particulate bone graft), which is a DMBM DMBM: Demineralized bone matrix xenograft.

The tooth root hereafter was known as the SS. An osteotomy was then sequentially prepared, and a 3.5 x 13 mm implant was inserted palatal to the SS (Figure 13). The jump gap was grafted with 100 mg of osseograft, which is a demineralized bone matrix (particulate xenogenic bone graft) (Figure 14).

**Figure 15:**
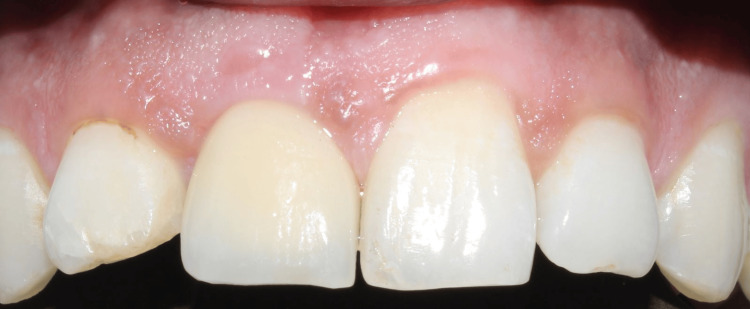
Case 2: Implant in relation to 11 was loaded after a period of eight months with a cement-retained crown

**Figure 16:**
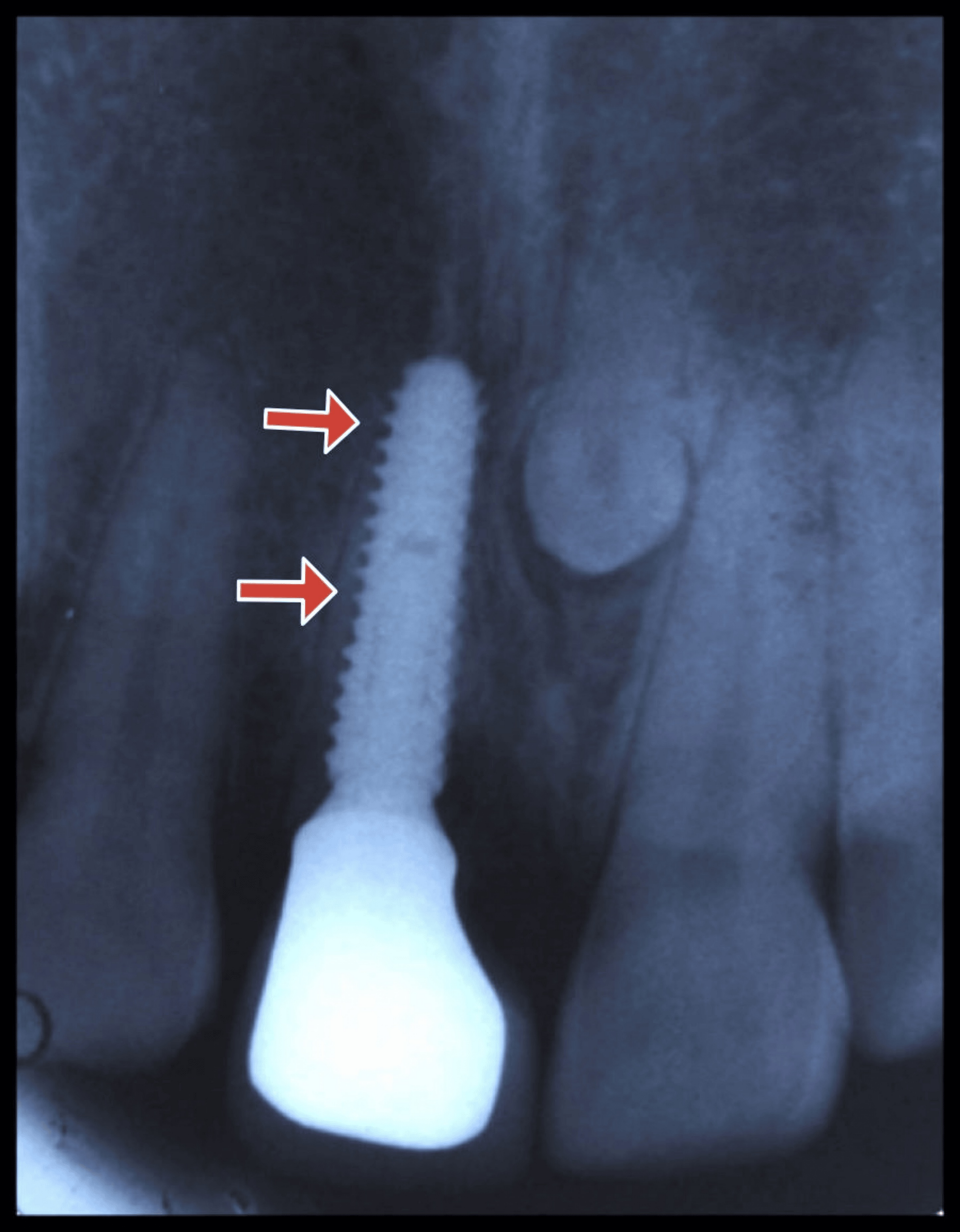
Case 2: During the follow-up visit one year after loading, IOPA was taken, which shows the intact buccal shield and a negligible amount of bone loss in relation to 11 IOPA: Intra-oral periapical radiograph.

After a healing period of eight months, the prosthetic phase was carried out as the patient reported with a successfully osseointegrated implant (Figure 15). A follow-up was done after three months, six months, and one year (Figure 16).

Case 3: Osseodensification

**Figure 17:**
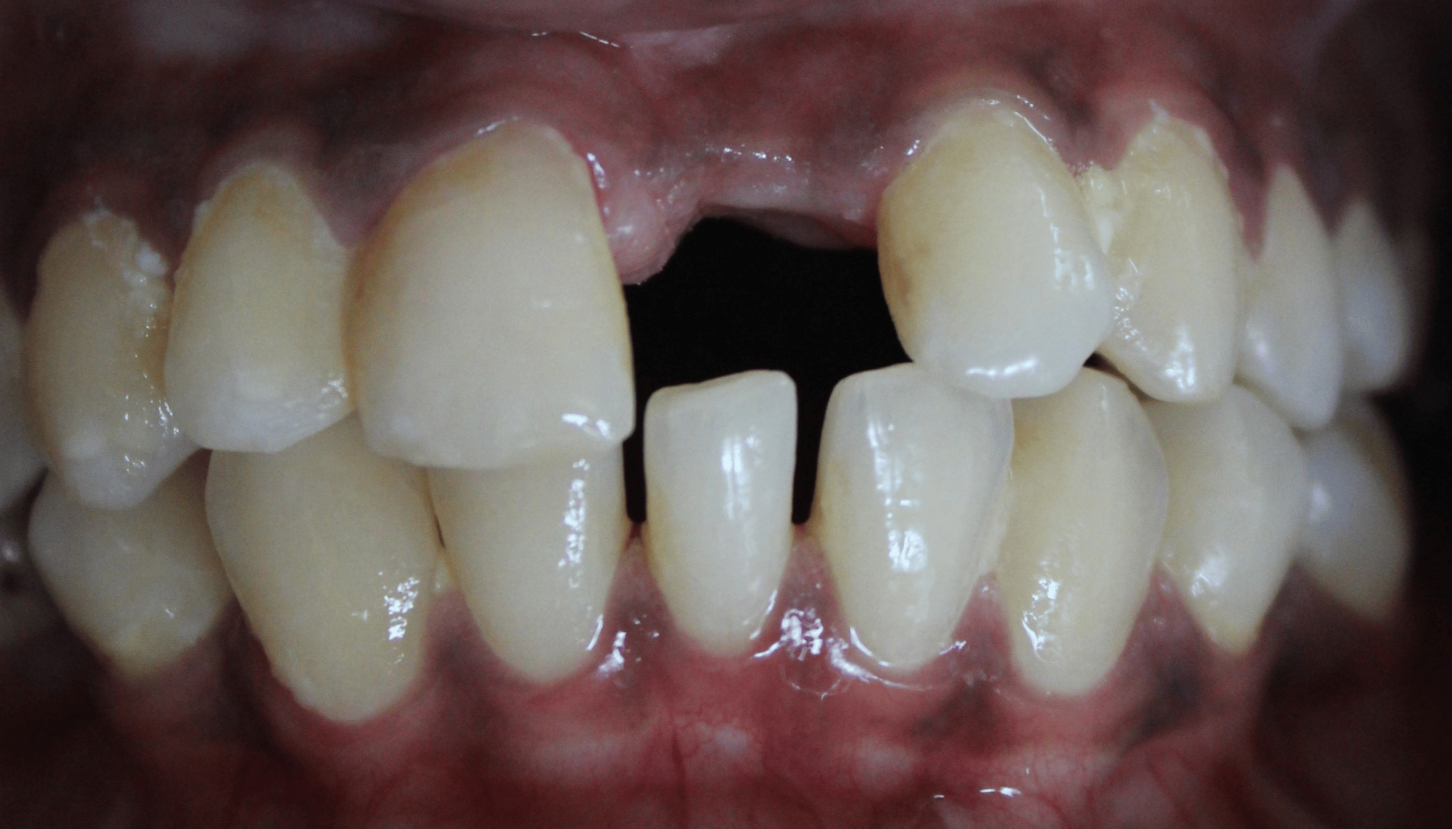
Case 3: Preoperative intra-oral image showing narrow edentulous space with respect to missing maxillary left central incisor with an adequate soft tissue thickness

**Figure 18:**
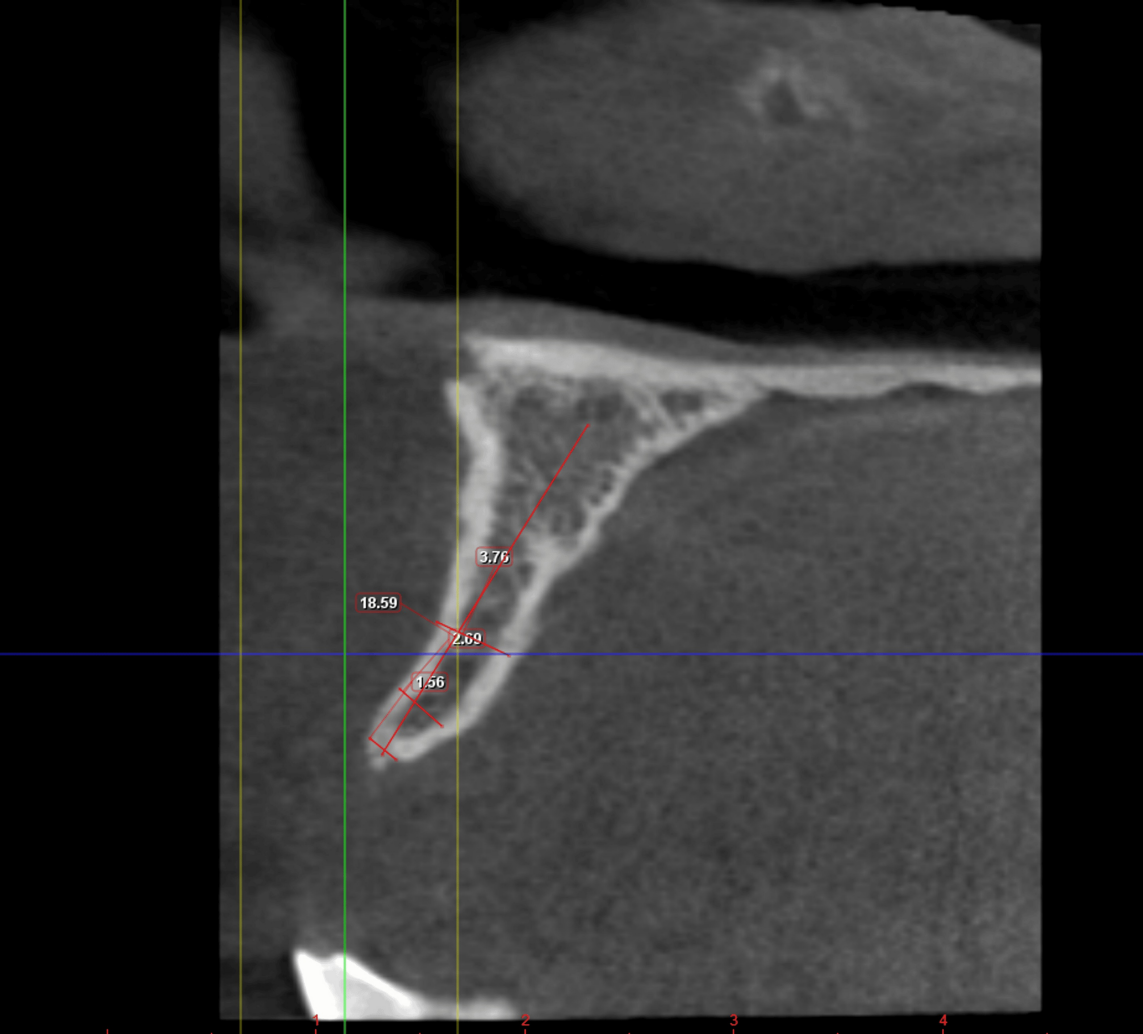
Case 3: The coronal section of the CBCT image of the patient showing 1.56 mm B-L width at the crest, 2.69 mm in the middle third, and 3.76 mm at the apical region of 21 region, which is inadequate for implant placement using conventional osteotomy B-L: Buccolingual.

A 24-year-old female reported for rehabilitation of a missing upper front tooth (Figure 17). On the evaluation of CBCT images, it was observed that the buccolingual width at the crest was inadequate (1.56 mm) and 2 mm from the crest - 2.69 mm (Figure 18).

**Figure 19:**
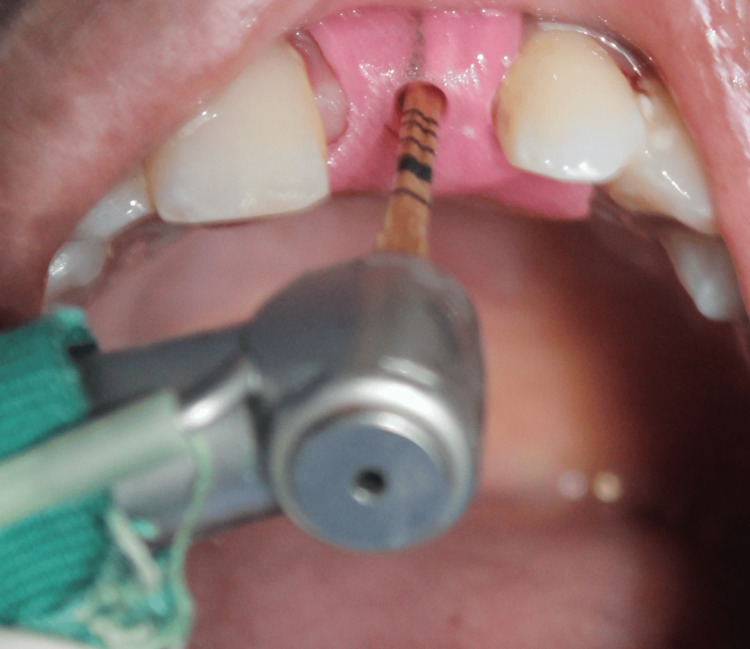
Case 3: Densah burs are used in counterclockwise rotation for the preparation of osteotomy to achieve bone condensation and improve primary stability

**Figure 20:**
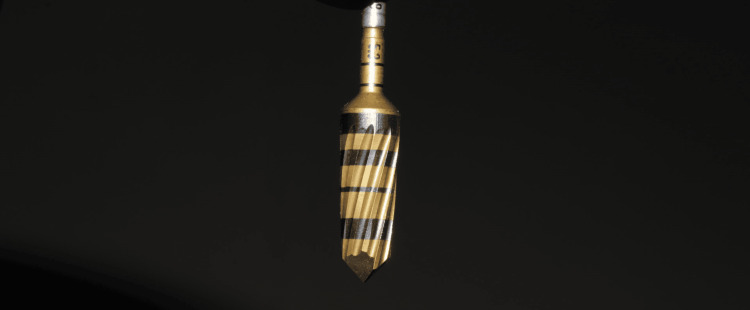
Case 3: Image depicting densah bur, which has more land than conventional straight drills that helps in controlled plastic deformation of bone

Recently, a novel method has been introduced for osteotomy preparation termed “osseodensification.” Since this technique allows bone expansion and improves primary stability, osseodensification using universal densah burs was preferred for this case. Osseodensification was preferred to combat narrow alveolar bone and less dense D3 bone evident from the CBCT images. Densah burs (Figures 19, 20) were used in a counterclockwise direction with increasing diameters from 2.0 to 2.5 mm, and an implant of 3 x 11.5 mm was placed.

**Figure 21:**
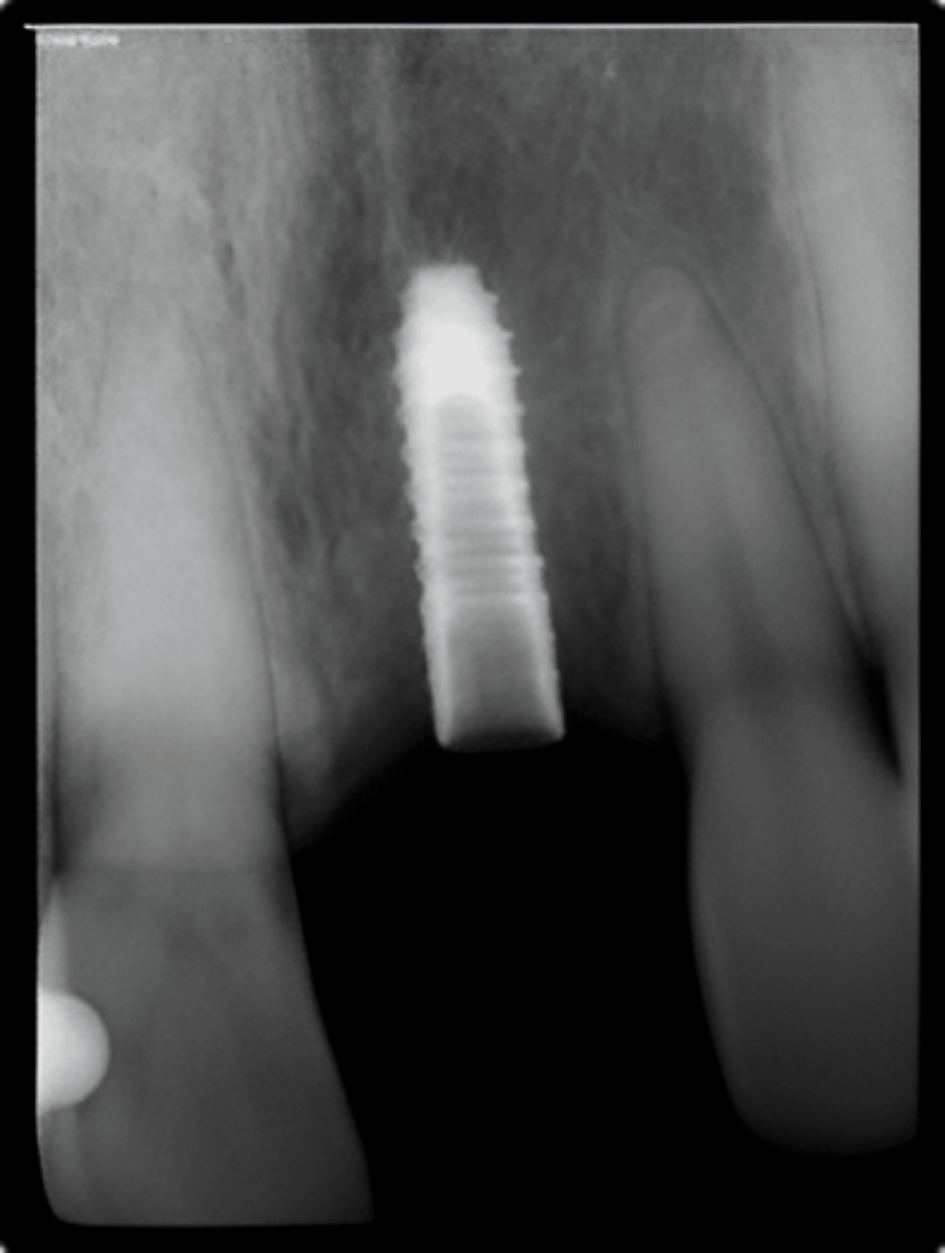
Case 3: Immediate postoperative IOPA depicting bone condensation with respect to 21 IOPA: Intra-oral periapical radiograph.

**Figure 22:**
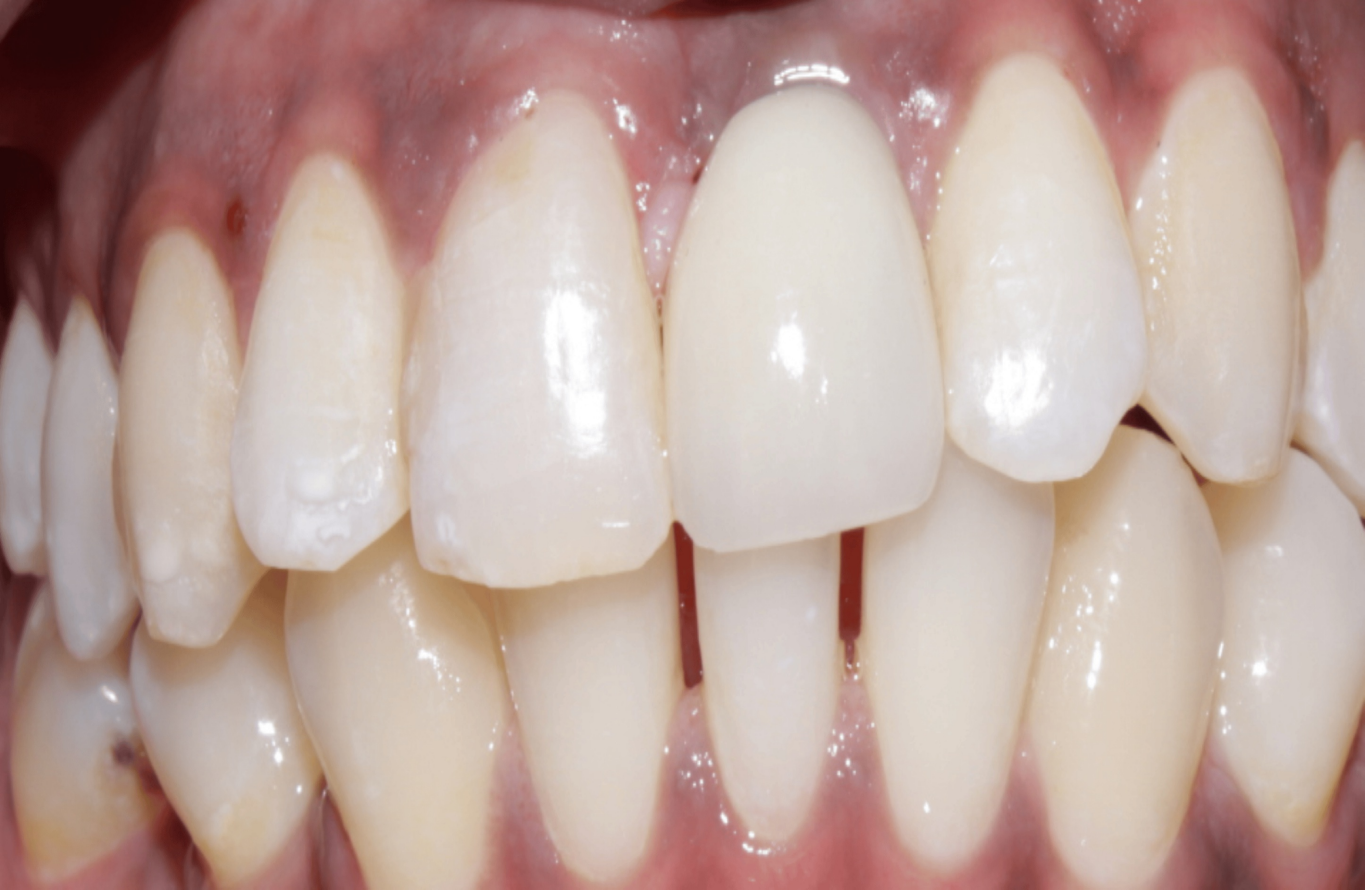
Case 3: Customized abutment was fabricated and torqued for 20 Ncm followed by luting of a cement-retained crown on the implant with respect to 21

**Figure 23:**
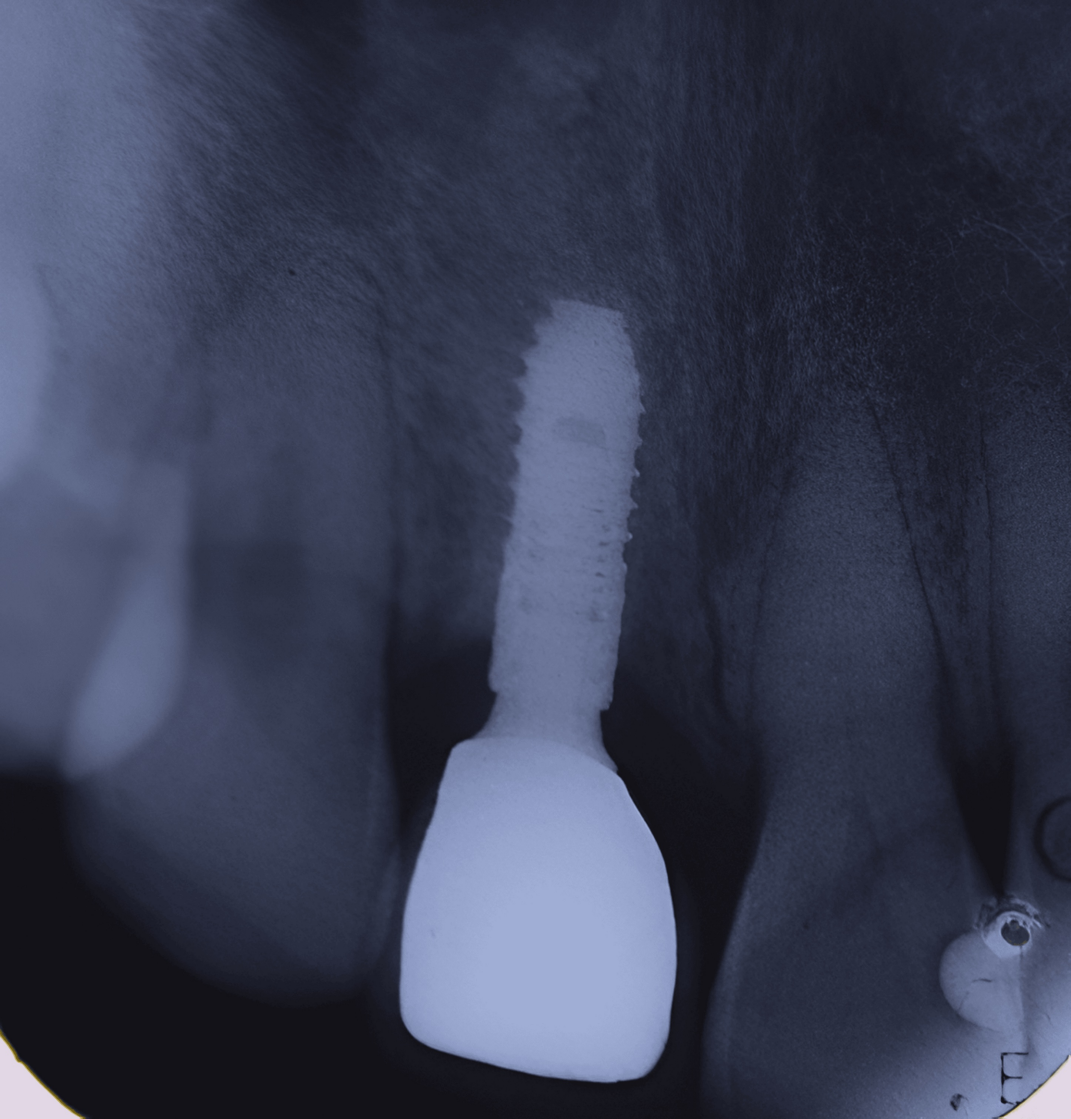
Case 3: IOPA taken during follow-up visit after six months showing successful functioning of the prosthesis without bone loss IOPA: Intra-oral periapical radiograph.

Immediate postoperative IOPA was taken to ensure bone condensation as depicted in Figure 21. It was noted during implant placement that the primary stability achieved was higher than that achieved using conventional osteotomy (implant stability quotient (ISQ) values were 70). After five months, the implant was loaded using a cement-retained prosthesis as shown in Figure 22. Follow-up was done after six months (Figure 23).

## Discussion

Endosseous dental implant placement using bone expansion and compaction, with or without adjunct bone grafting, is not uncommon. The technique of vertical bone compaction and elevation as proposed by Summers for sinus floor elevation with the use of osteotomes of increasing diameters has been well documented. Similar to Summers' technique, the lateral bone expansion kit utilizes bone-condensing burs and thread formers to achieve controlled, progressive expansion of bone [4]. Clinical advantages of this technique include maximum preservation of remaining bone and improvement of primary implant stability. Since these bone-condensing burs are used manually, the risk of overheating is minimized. The manual action guarantees major tactile sensibility and visibility [8,9]. At least a 3 mm width of the remaining bone crest is necessary to use this technique effectively, which is a major limitation. These techniques are not indicated in D1 and D2 bone as they can cause damage to microcirculation and can cause trabecular fractures [10]. In case 1, lateral bone expansion was chosen since the buccolingual bone width available was inadequate for a 3.5 mm diameter implant placement using conventional osteotomy. Using conventional osteotomy in these cases can result in perforation of the buccal bone.

SS technique involves the removal of the crown portion and palatal part of the root either up to the level of bone crest or 1 mm above while conserving the buccal fragment of the root intact, which will aid in the preservation of the supracrestal fibers with epithelial and connective tissue attachment [5]. Other ridge preservation techniques advocated in literature usually lessen the amount of ridge resorption but cannot obviate the loss of interdental bone and papillae. Maintenance of supracrestal fibers provides a great esthetic result due to their papilla preservation and better pontic site development. It has been postulated the normal events of physiological healing that result in resorption of the alveolar socket can be overcome as the retained fragment, which acts as a shield, averts the body from realizing that the tooth has been extracted. Crestal/buccal bone loss, exposure of the shield, and deep probing pockets are frequently encountered complications. There is a possibility of loss of SS by resorption or loss of buccal bone predisposing to implant exposure [11-13]. When patients present with an inadequate ferrule, the option of salvaging the tooth using post and core is ruled out. In situations similar to the case presented here, where there is a narrow labial bone, immediate implant after extraction can be risky as it can result in a fracture of the buccal bone. In such conditions, the SS technique can be opted to preserve the buccal bone aiding in immediate implant placement.

During conventional osteotomy techniques, the bone is subjected to microdamages resulting in compromised primary stability while delaying secondary stability due to the time required to repair the microdamages [14]. On the other hand, osseodensification is a procedure that improves the primary stability by revamping the bone chips that are collected on the surface during surgical preparation of the implant bed, resulting in an improved bone density encircling the implants. Osseodensification does not work in the cortical bone as it is nondynamic and lacks plasticity [15]. The need for at least 2 mm of trabecular bone to be performed could be considered a limitation of the osseodensification technique [16]. Systematic reviews and meta-analysis comparing conventional osteotomy versus osseodensification demonstrated that the osseodensification drilling protocol showed higher primary implant stability, secondary implant stability, and ridge width expansion in low-density bone and thin alveolar ridges than the conventional drilling protocol [17]; hence, this technique was opted in case 3.

Allogenic bone grafts have been established as a reliable alternative to autogenous bone with comparable clinical outcomes [18]. Bone augmentation techniques using synthetic graft materials are attractive alternatives to autografts because of their biocompatibility, better-handling properties, porosity, different dissolution rates, chemical and physical resemblance to bone minerals, and potentially unlimited supply at an economical cost.

## Conclusions

Rehabilitation of maxillary anterior teeth is a challenging task especially when a patient presents with bone defects. Bone augmentation using autogenous bone grafts followed by implant placement is the gold standard treatment for such patients to achieve desirable esthetics. But more often than not, patients may not be comfortable with the delay until the bone maturation. Hence, a few viable alternatives were brought into the forum such as lateral bone expansion using thread formers of increasing diameters, osseodensification using densah burs, and socket shield technique to preserve the existing root. The final esthetics also depends on the gingival biotype of the patient. This article presented cases with favorable gingival biotype but with reduced buccolingual bone width in the anterior esthetic zone restored using the above-mentioned techniques.
